# Bistable Switch in let-7 miRNA Biogenesis Pathway Involving Lin28

**DOI:** 10.3390/ijms151019119

**Published:** 2014-10-21

**Authors:** Fei Shi, Wenbao Yu, Xia Wang

**Affiliations:** 1 College of Veterinary Medicine, Sichuan Agricultural University, Ya’an 625014, Sichuan, China; E-Mail: dfeijtgf@gmail.com; 2 Department of Statistics, Seoul National University, Seoul 151-742, Korea; 3 School of Medical Engineering, Hefei University of Technology, Hefei 230009, Anhui, China

**Keywords:** let-7, Lin28, miRNA biogenesis, bistability

## Abstract

miRNAs are small noncoding RNAs capable of regulating gene expression at the post-transcriptional level. A growing body of evidence demonstrated that let-7 family of miRNAs, as one of the highly conserved miRNAs, plays an important role in cell differentiation and development, as well as tumor suppressor function depending on their levels of expression. To explore the physiological significance of let-7 in regulating cell fate decisions, we present a coarse grained model of let-7 biogenesis network, in which let-7 and its regulator Lin28 inhibit mutually. The dynamics of this minimal network architecture indicates that, as the concentration of Lin28 increases, the system undergoes a transition from monostability to a bistability and then to a one-way switch with increasing strength of positive feedback of let-7, while in the absence of Lin28 inhibition, the system loses bistability. Moreover, the ratio of degradation rates of let-7 and Lin28 is critical for the switching sensitivity and resistance to stimulus fluctuations. These findings may highlight why let-7 is required for normal gene expression in the context of embryonic development and oncogenesis, which will facilitate the development of approaches to exploit this regulatory pathway by manipulating Lin28/let-7 axis for novel treatments of human diseases.

## 1. Introduction

miRNAs comprise a large family of small RNA molecules that post-transcriptionally regulate gene expression in many biological pathways, such as proliferation, differentiation, and apoptosis [1]. Dysfunction of these precise processes, alone or in combination, is a key player in the emergence and progression of cancer, thus understanding the mechanisms that control both normal and deregulated miRNA expression may lead to new avenues for treatment of a variety of disorders [2,3].

**Figure 1:**
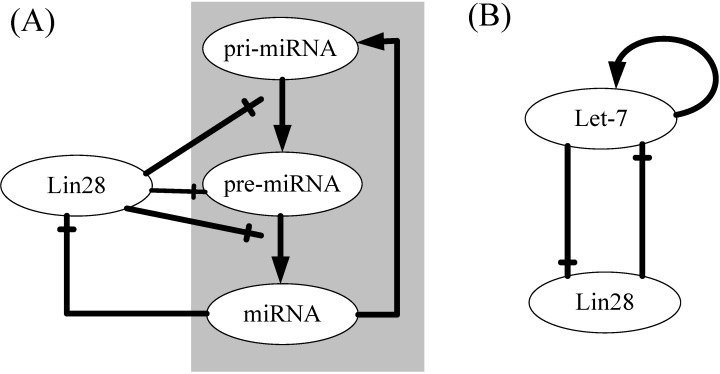
Schematic illustration of the let-7 biogenesis network involving Lin28. (**A**) Summary of the interactions among let-7 biogenesis network. All arrows refer to positive regulation of gene expression. The hammerheads refer to inhibition of translation or degradation of ncRNAs or miRNA processing; (**B**) Reduction of the model to an abstract one that keeps the essential structure of the network in **A**.

Typically, miRNA genes are initially transcribed by RNA polymerase II to generate long primary transcripts (pri-miRNA). Subsequently, pri-miRNA is processed by RNase-III enzymes Drosha into a ~60–100 nt hairpin structure termed the precursor-miRNA (pre-miRNA). Through the interaction with exportin-5 and Ran-GTP, the pre-miRNA is transported into the cytoplasm, where it undergoes a second round of processing catalyzed by Dicer. This cleavage event results in a double-stranded ~22 nt product [4,5]. One strand of the mature miRNA duplex is selectively retained in an Argonaute protein (Ago), the core component of the RNA-Induced Silencing Complex (RISC). Serving as guides in Ago complexes, mature miRNAs utilize imperfect base-pairing to recognize sequences in mRNA transcripts, leading to translational repression and destabilization of the target mRNAs in the cytoplasm [6,7].

let-7, an important member of the miRNA family, was originally identified in *C. elegans* and found to be conserved in controlling late temporal transitions during embryonic development across animal phylogeny [8]. It has gained fame owing to its well-appreciated regulatory mechanism involved in multiple biological processes of multicellular life [9]. Due to its functional importance, major progress has been made in understanding the basic mechanism of let-7 biogenesis. Recently, a study provided the first evidence that this miRNA can regulate its own biogenesis by directly targeting the 3' end of its own primary transcripts and promote downstream processing events, thus creating a positive feedback loop [10]. Besides, Lin28 is a small conserved cytoplasmic protein that contains two CCHC-type zinc fingers and a Cold-Shock Domain (CSD), both implicated in RNA binding and specifically expressed in embryonic cells [9,11,12]. It functions in blocking the processing of let-7 at both pri- and pre-miRNA steps [13,14], since Lin28 recruits terminal uridylyl transferase-4 (TUT4) to add uracil to the 3' end of pre-let-7, thereby resulting in blockade of let-7 maturation [15]. Interestingly, another recent study portrayed that *lin28* mRNAs are themselves let-7 targets, their expression are repressed by let-7, thus promoting neural stem cell differentiation [16]. These findings imply that a double-negative feedback loop is established between let-7 and Lin28 during cell differentiation (Figure 1A). This established the Lin28/let-7 axis which is highly conserved across the animal kingdom and nematode worms and operates as a switch function to maintain either a differentiated or an embryonic cell fate [9]. Despite concrete progress, a lot of mechanisms such as bistability and oscillations need to be further researched in a cellular system, which can help us to understand the crucial roles of let-7 in gene regulation and physiological functions.

Nonlinear phenomena of the interplay among noncoding RNA (ncRNA), mRNA and protein in cellular systems have been intensively investigated primarily through the study of a recurrent network motif mediated by ncRNAs [17,18,19,20,21,22,23]. These studies on the minimal architectures mediated by ncRNAs would greatly facilitate us to analyze complex systems assembled by these simple modules. Moreover, they promoted deep understandings of the ncRNA-mediated regulatory network motifs, which may also trigger interest in the bio-engineering or artificial control of specified components, interactions, and even network functions. In systems and synthetic biology, computational and theoretical tools are essential to perform *in silico* investigations of biological pathways, or novel synthetic circuits. In our previous work, we analyzed the generic miRNA biogenesis pathway without feedback loops and found three key rate-limiting steps as well as the suppression of “intrinsic noise” in this pathway [24]. Furthermore, we characterized the molecular dynamics mechanisms of how pre-miRNA is recognized and transported by exportin-5 and proposed a plausible mechanism of the pre-miRNA export cycle [25]. In this work, using a mathematical modeling of the minimal architecture abstracted from the complex miRNA biogenesis network (Figure 1B), we illustrate the mechanisms and conditions under which regulators operate. The model and the related discovery may be helpful for deep understanding of the regulation of miRNA biogenesis and for developing novel cancer treatments.

## 2. Results and Discussion

### 2.1. Model Introduction

The miRNA biogenesis pathway is a complex signaling network, which contains intertwined transcriptional controls, protease degradation, and other non-coding RNA regulations. We have previously modeled a miRNA biogenesis pathway without feedbacks [24]. To reduce network complexity, while keeping its essential regulatory features, our original model was simplified and two feedbacks were included. Figure 1 summarizes how the complex biogenesis network is coarse-grained to a model with two mutually inhibited components, which represent Lin28 and let-7. Unless otherwise noted, Lin28 is Lin28 protein; let-7 represents mature let-7. It is worth noting that, in the Lin28/let-7 feedback loop, we ignored the regulatory differences among members of Lin28 family (Lin28a and Lin28b) and members of let-7 family (let-7d, let-7f, let-7a, let-7b, let-7c, *etc.*). For let-7, transcription of the *let-7* gene is positively regulated by let-7 and can therefore be considered to act as an auto-regulatory positive feedback loop. This scenario is valid under conditions of relatively weak miRNA-target binding and large miRNA concentrations [23]. Thus we assume a Hill function ( $\frac{{\text{Γ}}_{1}{\left[let-7\right]}^{2}}{{\text{Γ}}_{2}+{\left[let-7\right]}^{2}\text{+}{\text{Γ}}_{3}[Lin28]}$ ) that was used in the model of E2F/Myc/miR-17-92 feedback loops [26] to represent the auto-regulated mode of let-7, which is inhibited by Lin28. The presence of [Lin28] in the denominator accounts for the Lin28-dependent down-regulation of let-7 biogenesis. The value of the parameter Γ3 is a measure of the efficiency of Lin28 inhibition of let-7 biogenesis, and it combines all factors that influence Lin28 to block the biogenesis of let-7. For Lin28, the efficiency of Lin28 mRNA degradation is associated with the binding of the let-7. Thus we assume a term ${\text{β}}_{P}[Lin28]\frac{\left[let-7\right]}{[let-7]+{\text{κ}}_{P}}$ for describing degradation of Lin28.

Finally, the dynamics of Lin28 and let-7 concentrations are respectively described by following Equations (1) and (2).

(1) $$\frac{d[Lin28]}{dt}={\text{α}}_{P}-{\text{β}}_{P}[Lin28]\frac{\left[let-7\right]}{[let-7]+{\text{κ}}_{P}}$$

(2) $$\frac{d[Lin28]}{dt}={\text{α}}_{P}-{\text{β}}_{P}[Lin28]\frac{\left[let-7\right]}{[let-7]+{\text{κ}}_{P}}$$

where [*Lin*28] and [*let* − 7] represent the concentrations of Lin28 and let-7, respectively. α*_M_* denotes expression of let-7, α*_P_* describes the constitutive Lin28 expression due to signal transduction pathways activated by signals in the extracellular medium. β*_P_* and β*_M_* denote the degradation rates of Lin28 and let-7, respectively. κ*_P_* is the coefficient of Lin28 expression inhibited by let-7. Γ_1_ is the coefficient of let-7 expression, Γ_2_ is the rate constant of let-7 expression, and Γ_3_ is a measure of the efficiency of let-7 expression inhibited by Lin28.

After a nondimensionalizing processes, Equations (1) and (2) can be rewritten as follows:

(3) $$\frac{d\text{ψ}}{d\text{τ}}=1-\phi \frac{\text{ψ}}{\text{ψ + γ}}$$

(4) $$\frac{d\text{ψ}}{d\text{τ}}=\text{ε}(\text{α}+\frac{{\text{γ}}_{\text{1}}{\text{ψ}}^{\text{2}}}{{\text{γ}}_{\text{2}}{\text{+ ψ}}^{\text{2}}{\text{+ γ}}_{\text{3}}\phi }-\text{ψ})$$

where $\phi ={\text{β}}_{P}[Lin28]/{\text{α}}_{P}$, $\text{ψ}={\text{β}}_{M}[let-7]/{\text{α}}_{M}$, $\text{τ}={\text{β}}_{P}t$, $\text{α}={\text{α}}_{M}/{\text{α}}_{P}$, ${\text{γ}}_{1}\text{=}{\text{Γ}}_{1}/{\text{β}}_{P}$, $\text{γ}=\kappa_{P}{\text{β}}_{M}/{\text{α}}_{P}$, $\text{ε = }{\text{β}}_{M}/{\text{β}}_{P}$, ${\text{γ}}_{2}\text{=}{\text{Γ}}_{2}{\text{β}}_{M}^{2}/{\text{α}}_{P}^{2}$ , and ${\text{γ}}_{3}\text{=}{\text{Γ}}_{3}{\text{β}}_{M}^{2}/{\text{α}}_{P}{\text{β}}_{P}$ . Thus, γ, γ_1_, γ_2_ and γ_3_, respectively, represents the coefficient of Lin28 expression inhibited by let-7, the coefficient of let-7 expression, the rate constant of let-7 expression, and the efficiency of let-7 expression inhibited by Lin28, after nondimensionalization. Since half-life of let-7 after Tamoxifen (TAM) treatment is about 4 h [27], and half-life of Lin28 is about 1.5 h [28], thus ε is about 0.4. α is allowed to vary in the range of 0~0.4. γ_1_ and γ_3_ vary from 2.0~5.0 and 0~2.5 respectively [26,29,30]. γ_2_ is set as 1.0.

### 2.2. Steady States of the Model

**Figure 2:**
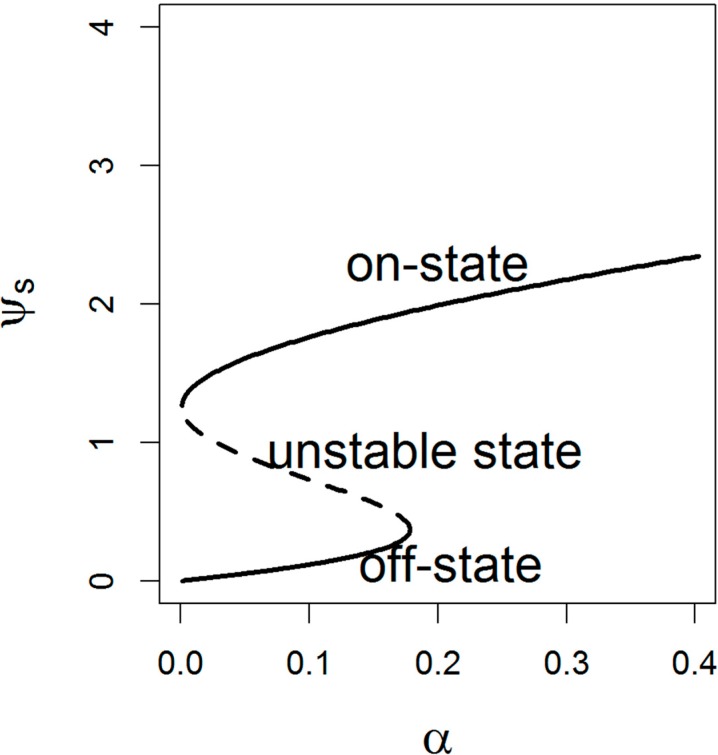
The dimensionless let-7 concentration ψ_s_ as a function of α under steady state conditions. The solid and dashed lines correspond respectively to the stable and unstable steady state (α = 0.01~0.17). Here γ = 0.1, ε = 0.02, γ_2_ = 1, γ_3_ = 0.5.

To study the effect of regulatory motifs on ordering of the events, we have computed the overall steady state response of the system, which is given as a signal response curve. For steady state, we set Equations (3) and (4) in Experimental Section equal to zero and solved for the roots of the algebraic equations from the right-hand sides, then a relation between ψ_s_ and *ϕ*_s_ was obtained:

(5) $${\text{ψ}}_{\text{s}}=\frac{\text{γ}}{\phi_{\text{s}}-1}$$

where ψ_s_ and *ϕ*_s_ represent the steady states of ψ and *ϕ*, respectively. A steady state is a situation in which all state variables are constant in spite of ongoing processes that strive to change them. According to this equation, the steady states of let-7 and Lin28 increase or decrease in the opposite direction. This model prediction is consistent with observations in many normal and cancer (or differentiated) cells that expression levels of let-7 and Lin28 are reciprocal [3,31,32]. Since it is one of the factors required for pluripotency of cells, let-7 involved in a regulatory feedback loop with Lin28 is essential for stem cell differentiation and maintenance [33,34]. Compared with normal tissues, global miRNA levels are usually depressed in tumor samples, suggesting that a key role for miRNA in maintaining differentiated cell phenotypes and deregulation of miRNA expression or biogenesis implicated in a various diseases. For examples, increased biogenesis of let-7 in differentiated cells represses progenitor cell-specific mRNA to increase the fidelity of cell fate transition during differentiation [3,34], while exogenous Lin28 rescued the neural precursors (NPCs) proliferation and some neurogenic deficits in the absence of SOX2 [34]. Analysis of tissue and cultured cell lines indicated that Lin28 was predominately expressed in undifferentiated embryonic tissue and stem cells, and was very low in differentiated cell and tissue types such as fibroblast cells, hepatic and neural tissue [35]. Therefore, for convenience, we assign the lower/higher let-7 concentration as the off/on state. Generally, the on-state of let-7 denotes early development where Lin28 is expressed at a very low level, the off-state represents other processes (e.g., cancer) where Lin28 is over-expressed. Notably, the range of ψ_s_ that defines the off/on state is chosen arbitrarily; one would expect that the range of the off/on state is dependent on the specific cellular system and on the perturbations of the system that drives it toward an on/off state.

let-7 activity presents discontinuous bistable behavior with respect to inhibition of Lin28 (Figure 2). The S-shaped region of the response curves indicates that for each inhibition value of Lin28 the systems have two possible stable steady states separated by an unstable state (α = 0.01–0.17). The higher stable state denotes the let-7 level in an on-state, in which increased let-7 levels reduce proliferation and lead to the decrease of several oncogene targets including Myc [36].

It can be observed that the let-7 activity occupies a higher stable steady state until the inhibition of let-7 by high concentration of Lin28 reaches the limit point of stable and unstable steady states. At this critical threshold, the higher stable state of let-7 switches to the lower stable state (off-state), which is accompanied by the upregulation of Lin28. This suggests that the Lin28/let-7 pathway plays an important role in fine-tuning cellular processes of self-renewal and differentiation and Lin28A/B upregulation in some cases expression correlates with advanced tumor stage and poor prognosis [9].

### 2.3. Effects of the Positive Feedback of let-7 on Switching Behavior

**Figure 3:**
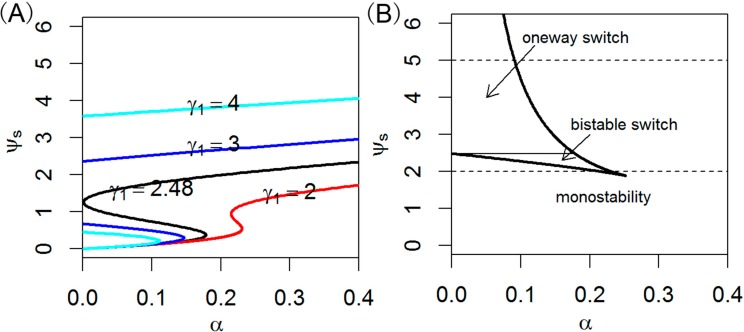
Steady-state bifurcation diagrams of the ψ_s_ (**A**) and phase diagrams (**B**) of switching behavior. Here γ = 0.1, ε = 0.02, γ_2_ = 1, γ_3_ = 0.5.

miRNAs are important components embedded in gene regulatory networks and are found to establish different kinds of network motifs with their transcription factors and targets. Here, we investigate the features of this well-constructed let-7 mediated network motif on several parameters. Since module let-7 with a positive feedback loop can create switching behavior, it has two possible stable states in the appropriate parameter regime. Here, we probe steady-state ψ_s_ bifurcation diagrams as a function of the bifurcation parameter α for different values of γ_1_ which are shown in the left panels of Figure 3. Taken physiological constraints into consideration, the horizontal axis in Figure 3 extends from 0 to some maximum value of α. To create similar experimental curves, one can measure the miRNA levels when (normal and cancer) cells grow under different experiment conditions. From Figure 3, we find that ψ_s_ increases as γ_1_ increases for a given α and γ_3_. The whole phase diagrams of switch behaviors including bistable and one-way switches, and a monostable region for different γ_3_ values is explored. With increases of γ_1_, the system undergoes a transition from monostability to a bistable switch and then to a one-way switch. The parameter α exhibits a similar influence.

When 2 ≤ γ_1_ < 2.48 in Figure 3A, as α increases along the lower stable branch, ψ_s_ remains in the off-state until α = 0.17. With α further increases, the off-state is lost, and ψ_s_ switches to the upper stable state, the on-state. Thus the system shows bistable switch behavior and hysteresis, meaning that the stimulus must exceed a threshold to switch the system from one steady state to another, and it may remain there when the stimulus decreases. Although this predicted result has not been validated by experimental methods, it might also be a desirable property for such a system because the Lin28/let-7 axis is central to maintaining proper cell fate and coordinating proliferation, growth, and energy utilization at the cellular level as well as growth, developmental timing, tissue homeostasis, and metabolism in whole organisms [9].

When γ_1_ ≥ 2.48 in Figure 3A (e.g., γ_1_ = 3, 4), a portion of the S-shaped response curve of let-7 is pushed onto the negative regime of the diagram therefore the off-state threshold of let-7 is a negative number. This suggests that very high level of let-7 may interrupt the Lin28/let-7 feedback circuit, and disables the cell to maintain and transmit its state. As the α decreases, the activity of let-7 moves toward the left of the bifurcation diagram along the upper stable state and remains in an on-state when α equals to zero. It indicates that the system acts as an irreversible one-way switch. Note that, when we use “on” and “off” state, the level of let-7 (ψ_s_) or the coefficient of let-7 expression (γ_1_) used to correlate with entry into or exit from a certain state needs to be clearly specified. However, we should keep in mind that experimental determination of γ_1_ would be more difficult in practice compared with measuring let-7 levels, and therefore, qualitative classification using changes in let-7 levels is commonly used [26].

### 2.4. Effects of Dual Negative Feedback Regulation of Lin28 and let-7 on Switching Behavior

It is well known that Lin28 can inhibit let-7 maturation and let-7 can inversely repress Lin28 expression. This Lin28/let-7 axis has been demonstrated to play central roles in cell differentiation and development [9]. We perturbed the inhibition of let-7 by Lin28 to study the contribution of the double negative feedback loop on the overall response curves of let-7.

**Figure 4:**
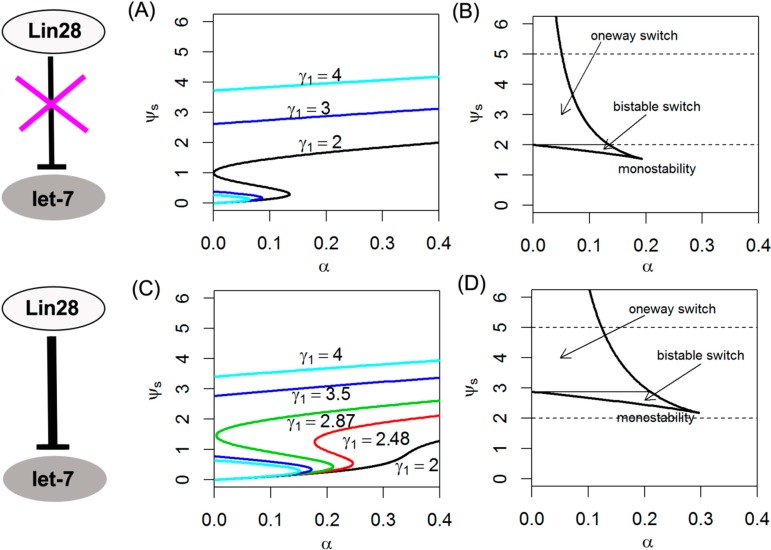
Steady-state bifurcation diagrams of the ψ_s_ and phase diagrams of switching behavior. In (**A**) and (**B**), γ_3_ = 0, γ = 0.1, ε = 0.02, γ_2_ = 1; in (**C**) and (**D**), γ_3_ = 1, γ = 0.1, ε = 0.02, γ_2_ = 1.

Firstly, we delete the Lin28 inhibition (γ_3_ = 0) (Figure 4A), the results show that the absence of inhibition by Lin28 slightly change the S-shape of the let-7 response curve compared with γ_3_ = 0.5, it pushes the off-state threshold of let-7 to the left on the diagram. Thus, it decreases the switch region enclosed in the effective domain of γ_1_ (from 2.0 to 5.0, represented by two dashed lines). For example, when 2 ≤ γ_1_ < 2.48, it pushes a portion of the S-shaped curve further into the negative regime, which acts as an irreversible one-way switch (Figure 4A). Notably, as the strength of the positive feedback γ_1_ increases (from 2.0 to 5.0), the system only undergoes a monostability; the parameter α takes a similar effect (Figure 4A,B).

Then, we increase the strength of inhibition by Lin28 (γ_3_ = 1) (Figure 4C), it also maintains the S-shape of the let-7 response curve compared with γ_3_ = 0.5, while it pushes the off-state threshold of let-7 to the right on the diagram (Figure 4C). Therefore the regime of γ_1_ is enlarged to keep the bistability of let-7. For instance, when 2.48 ≤ γ_1_ < 2.87, the system still has bistable switching behavior (Figure 4C). Besides, the threshold that makes bistability of let-7 switch to monostability is also increased (Figure 4D).

**Figure 5:**
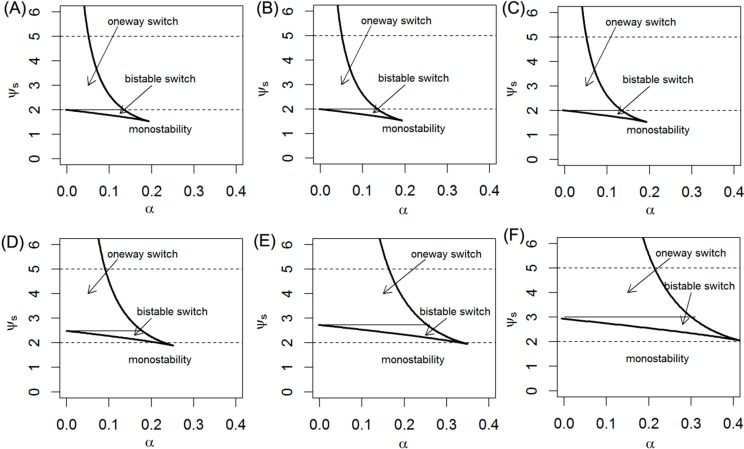
Phase diagrams of switching behavior. (**A**) γ = 0.1, γ_3_ = 0; (**B**) γ = 1, γ_3_ = 0; (**C**) γ = 2, γ_3_ = 0; (**D**) γ = 0.1, γ_3_ = 0.5; (**E**) γ = 1, γ_3_ = 0.5; (**F**) γ = 2, γ_3_ = 0.5. The other parameters ε = 0.02, γ_2_ = 1.

In addition, we investigate the effects of let-7 inhibitory strength (γ) on switching behavior. If the Lin28 inhibition (γ_3_ = 0) were deleted, γ has no effect on let-7 response curves (Figure 5A–C), and the systems display only the one-way switch. As α increases, the systems almost stay in the on-state (*In vivo*, the probability of switching from one status to another is also very low). In the presence of Lin28 inhibition, the effective region of the bistable switch is expanded by increasing the strength of γ (Figure 5D–F). In the case of small α values, the system generates diverse dynamical behaviors.

### 2.5. Effects of Expression of Lin28 and let-7 on Switching Behavior

**Figure 6:**
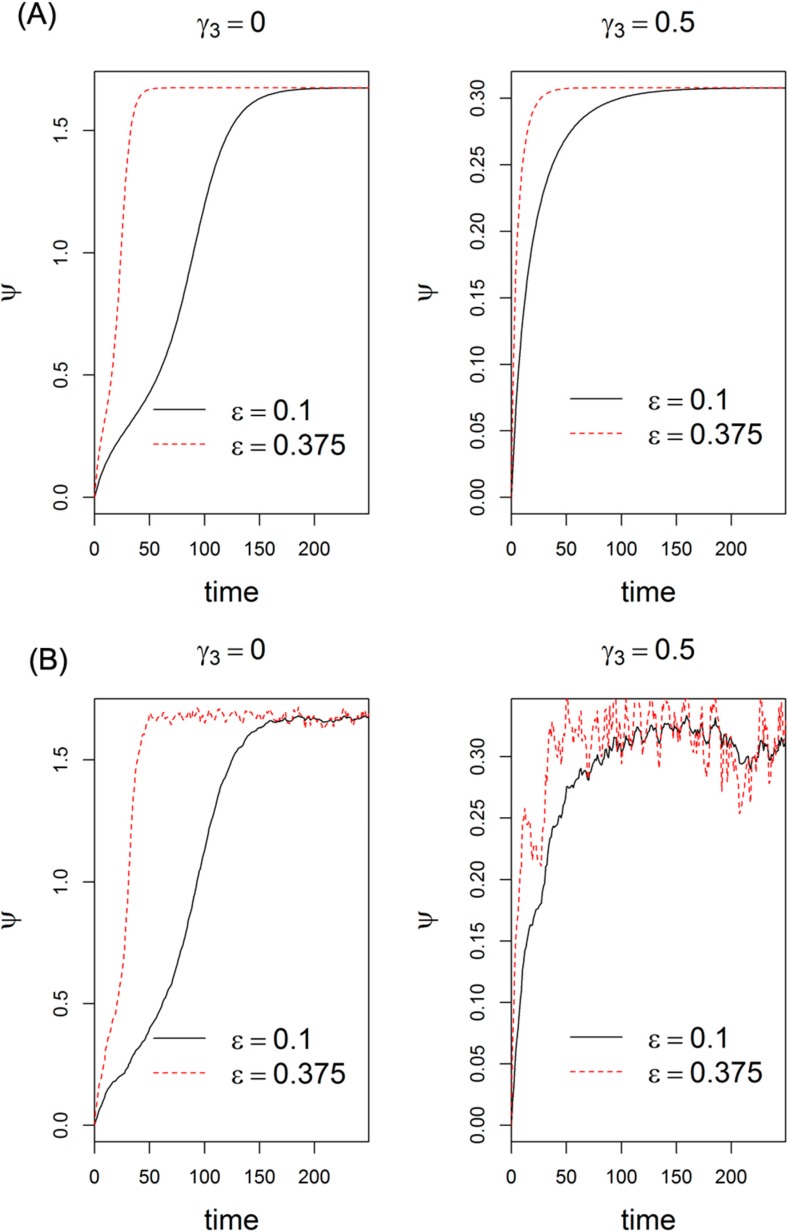
Dynamic behaviors of let-7 in response to different ε and α. (**A**) The dynamic behaviors of let-7 in response to ε = 0.1 and 0.375; (**B**) The dynamic behaviors of let-7 in response to environment fluctuation described by Gaussian white noise with mean α = 0.2 and variance 0.05.

We note that ε = β_*M*_ /β_*P*_ represents the ratio of the degradation rates of let-7 and Lin28, α = α_*M*_ /α_*P*_ denotes the ratio of the expression rates of let-7 and Lin28. Both dimensionless parameters determine the steady states of let-7 and Lin28, and affect the switch behavior. Although mRNAs are generally less stable than many proteins, this is not the case for miRNAs, which are up to 10 times more stable than mRNA [37], for example, let-7 [37] is more stable than Lin28 [38]. Thus, the values of ε employed here are less than one. The dynamic behaviors of let-7 in response to ε = 0.1 and 0.375 are illustrated in Figure 6A. From the figure, we find that as the value of ε increases, let-7 undergoes a faster transition from off to on state. This phenomenon is independent of Lin28 inhibition (γ_3_). That is, the value of ε is critical for the switching sensitivity. For examples, when ε = 0.1 in Figure 6A (γ_3_ = 0), the system is initially in the off-state, and then rises to 1.7, the on-state of the system, at time *t* = 200. When ε = 0.375, the system jumps to on state at *t* = 40. It has been reported that *Lin28* mRNA expression can be depressed by several other miRNAs including miR-125, miR-9, and miR-30 [39] and miR-181 [33], and Lin28 expression can be modulated by proteasome inhibitors such as MG132 [40]. This result suggests that the posttranscriptional modification of Lin28 activity during let-7 biogenesis and the interruption of Lin28/let-7 axis may play a central role in carcinogenesis.

Gene expression in cellular processes is essentially stochastic due to the variation in the number of gene copies and random fluctuations in environment [41]. Gene expression noise can be decomposed into “intrinsic” and “extrinsic” noise according to the sources [41]. Specifically, fluctuations arising from the inherent stochastic nature of biochemical reactions associated with transcription, translation, mRNA and protein degradation commonly refer to “intrinsic” noise; the variability induced by fluctuating environment and cell-to-cell variations can be classified as “extrinsic” noise. The “intrinsic” noise properties in miRNA-mediated gene network has been explored in our previous work [24] and others [23,42], it was found that miRNAs could buffer against fluctuations arising from intrinsic stochasticity of biochemical reactions, imparting precision and robustness to regulation of gene expression. Thus, in this section, we merely analyzed the “extrinsic” noise properties of the network. We provide the dynamic behaviors of let-7 in response to environment fluctuation described by Gaussian white noise with mean α = 0.2 and variance 0.05 (Figure 6B). The trends of temporal expression profiles of let-7 in fluctuation cases are similar as those in the cases without fluctuation. Increasing value of ε would enhance the amplitude of let-7 fluctuation, suggesting that large ε value decreases the stability to resist stimulus fluctuations. Similarly, an experimental observation on miR-7 has been demonstrated in *Drosophila*, where the miR-7 is required for normal gene expression and sensory organ fate determination under fluctuating temperature conditions [43] by regulating the levels of its downstream target, *yan*. In addition, although gene regulatory processes including Lin28/let-7 axis are typically subject to considerable delays induced by the underlying biochemical reactions, the impacts of a time delay would probably not qualitatively change the results of a negative feedback loop [44,45]. Combined the previous results of “intrinsic” noise in miRNA-mediated gene network [23,42], miRNA appears to be resistant to both “intrinsic” and “extrinsic” noise.

### 2.6. Implications of Lin28/let-7 Axis in Cancer Treatment

let-7 is widely viewed as a tumor suppressor miRNA and its expression is downregulated in many cancer types compared to normal tissue during tumor progression. Precise regulation of let-7 by Lin28 is a rapidly growing field and it points to the importance of small RNA metabolism in disparate fields of mammalian biology [9]. Our model of let-7 regulated by Lin28 may provide insights into understanding of how precise levels of let-7 are maintained in the context of cell development and oncogenesis, which would facilitate the development of approaches to exploit this regulatory pathway by manipulating Lin28/let-7 axis for novel treatments of human diseases. For example, miR-181 upregulates expression of let-7 by effectively repressing Lin28 expression, and eventually promoting megakaryocytic differentiation, thus providing insight into future development of miRNA-oriented therapeutics [33]. Therefore, our results suggest that deactivation of Lin28 in some cancer cells may greatly enhance the let-7-dependent tumor suppression and improve the treatment efficiency.

## 3. Experimental Section

All numerical bifurcation analyses of the ordinary differential equations were performed with OSCILL 8.28 [46]. In addition, to simulate the cellular processes which take place in a fluctuating environment [41], a small perturbation in the stimulus input (“extrinsic noise”) is also involved. In other words, we also studied the ordinary differential equations where α in Equation (4) follows a Gaussian distribution with mean 0.2 and variance 0.05. All differential equations were numerically solved by the Runge–Kutta method by R software (2013) [47].

## 4. Conclusions

By systematically analyzing the coarse grained model of let-7 biogenesis network in close association with plausible experimental parameters, we find that, in the presence of Lin28 inhibition, the system undergoes a transition from monostability to a bistability and then to a one-way switch as strength of positive feedback of let-7 increases, while in the absence of Lin28 inhibition, the system loses bistability. Moreover, the ratio of degradation rates of let-7 and Lin28 is critical for the switching sensitivity and resistance to stimulus fluctuations. These findings may highlight why let-7 is required for normal gene expression in the context of cell development and oncogenesis, facilitating development of approaches to exploit the regulatory pathway by manipulating Lin28/let-7 axis for novel treatments of human diseases. Furthermore, the abstract model developed here could be further integrated by including additional signaling pathways that are associated with human diseases.
